# Remote collaboration in virtual reality induces physiological synchrony comparable to face-to-face interaction

**DOI:** 10.1038/s41598-026-35955-y

**Published:** 2026-01-27

**Authors:** Stephan Streuber, Sarah Rogula, Maria Alejandra Quirós-Ramírez, Jens Pruessner

**Affiliations:** 1 University of Konstanz, Department of Psychology, 78464 Konstanz, Germany; 2 Coburg University of Applied Sciences, Electrical Engineering and Computer Science, 96450 Coburg, Germany; 3 Department of Politics & Public Administration, University of Konstanz, Konstanz, Germany; 4 Centre for the Advanced Study of Collective Behaviour, University of Konstanz, Konstanz, Germany

**Keywords:** Multi-user virtual reality, Embodied virtual reality, Heart rate variability, Physiological synchrony, Social interaction, Creativity, Social cohesion, Cooperation, Human behaviour

## Abstract

Physiological synchrony refers to the temporal alignment of bodily signals, such as heart rate variability, between two or more individuals during social interaction. It reflects implicit, often unconscious processes that arise when people share attention, emotions, or behavioral rhythms in close physical proximity. Because these coordinated physiological patterns are linked to social cohesion, rapport, and effective communication, physiological synchrony provides a valuable window into the quality and dynamics of social interaction. Here, we study physiological synchrony during virtual interaction where interaction partners are not physically co-located but remotely connected via technology. This allows us to capture aspects of social connectedness that are not accessible through self-report or behavior alone, making it a powerful tool for understanding how people engage and collaborate across different media. In our study, triads of participants performed a collective creativity task in one of three conditions: face-to-face (F2F) collaboration, remote collaboration using video conferencing (Video), or remote collaboration using immersive Virtual Reality (VR). To quantify social interaction quality, we measured creative group performance, social presence, and heart rate variability synchrony (HRVS) as a marker of social cohesion. As expected, creative group performance and social presence were highest in the F2F condition and significantly reduced in the VR and Video conditions. However, we observed strong HRV synchrony in the VR and F2F conditions and significantly weaker HRV synchrony in the Video condition. Our study supports the idea that VR (unlike video conferencing) supports physiological synchronization processes important for social interactions. Future studies need to identify the underlying physiological and psychological processes.

## Introduction

Humans are social beings, and social interactions shape our lives and brains. With the increasing prevalence of technology in our lives, many social interactions are now taking place in digital spaces and virtual environments, where we interact with others remotely rather than in person. The digitalization of social interactions affects all areas of our daily lives, including work, education, and personal relationships. A prominent example is the transformation of work practices in global organizations and international companies through the increasing use of virtual teams, in which geographically dispersed members collaborate via digital communication technologies^1^. By removing temporal and spatial barriers, digital technologies enable organizations to bring together diverse talents and expertise from across the globe^2^. COVID-19 further accelerated a shift towards virtual teams by normalizing large-scale remote work^3^. Several theoretical frameworks have been developed to explain the factors that determine the success and performance of virtual teams (for a review, see^2,4^). Most of these frameworks focus on the importance of communication, trust, and social presence in virtual collaboration.

Media Richness Theory^5^ posits that communication media vary in their capacity to convey information effectively. Media richness is defined by the availability of multiple communication channels (e.g., textual, vocal, visual), the immediacy of feedback, and the extent to which a medium supports social presence. Richer media are thought to facilitate collaboration by enabling mutual understanding, building trust, and supporting shared cognition^4^. In line with Media Richness Theory, studies found better communication and creative team performance in face-to-face (F2F) interaction compared to video conferencing^3,6^, and better team performance in video conferencing than in chat interfaces^7^. Another line of research investigates the effects of digital communication on creative group performance of virtual teams. Creativity is at the heart of discovery and innovation, and has been regarded as one of the most critical skills in employees^6^. Pooling diverse perspectives and skills through virtual teams applications enables convergent and divergent thinking, which are regarded as crucial for creativity^8^. On the contrary, digital communication might curb creative group performance by narrowing cognition^3^. In line with this, Brucks et al. (2022) found that teams are less creative when communicating via video conferencing tools compared to face-to-face interaction^3^. Taken together, these findings indicate that the level of media richness in computer-mediated communication (CMC) might significantly influence social cohesion and creativity in virtual teams.

New communication media that support high degrees of media richness are (social) Virtual Reality (VR) platforms such as Meta Horizon Worlds (https://www.oculus.com/facebook-horizon/), VIVE Sync (https://sync.vive.com/) or Spatial (https://www.spatial.io/). These allow users to meet in VR remotely from any place in the world using consumer hardware such as head-mounted displays.^9,10^. Motion-tracking technologies enable the inclusion of additional body parts in the simulation, such as hands, faces, and bodies, so that users can perceive and interact with each other’s avatars. Unlike video conferencing or other forms of computer-mediated communication, VR creates the illusion of co-presence (or social presence), which is the degree to which participants perceive and treat other avatars as real people^11^. It has been shown that VR creates higher social presence^12,13^ than video conferencing, and that higher social presence scores lead to conversation patterns more similar to face-to-face interaction^14^. Another advantage of VR is that it allows for natural interaction (e.g., turn-taking during a conversation, mutual gaze, proxemics) and the exchange of verbal and non-verbal cues (e.g., pointing and hand gestures)^15,16^. Verbal cues are considered important for the transmission of explicit information. Non-verbal cues are important for the transmission of emotional states and for regulating the flow and pace of the interaction^17–19^. Given its capacity to facilitate naturalistic social interactions, VR can be a powerful tool for fostering trust, fostering creativity, and strengthening social cohesion. However, experimental evidence regarding its effectiveness compared to video conferencing or other communication media remains mixed^20,21^. This inconsistency may, in part, reflect variability in the tasks employed and the measurement tools used to assess the impact of different communication media on social interaction.

One promising indicator of social cohesion and interaction quality in both real and virtual teams is interpersonal synchrony: the alignment of biobehavioral processes between several individuals^22^. Synchrony is a pervasive, often spontaneous, and effortless phenomenon observed in diverse social contexts, including human interactions. It appears as a “unifying principle” in complex, self-organizing systems, ranging from pendulum clocks to the human brain. In human interaction, synchrony can arise from mutual interactions where individuals dynamically adjust to each other, rather than simply responding independently to external stimuli^23^. Mathematical models show that interpersonal synchrony simplifies social interactions by reducing the degrees of freedom within a dyad or group, reducing the cognitive burden, and allowing individuals to chunk complex interactions into fewer, more manageable pieces. Synchrony serves multiple, interconnected, and often adaptive functions in human social interactions, spanning cognitive, social, and emotional domains. It reduces the cognitive burden and increases the predictability of social interactions, making it easier for individuals to anticipate each other’s behaviour and reducing prediction error. It facilitates information flow and helps establish shared mental representations or “common ground” (e.g., common language, shared subjective experiences), which is crucial for successful communication and joint problem-solving. As such, it can help individuals function as a cohesive unit to achieve shared goals, which is essential for many collaborative activities. Finally, synchrony serves as a powerful signal of interpersonal similarity, attunement, rapport, and prosocial intentions. It can act as a “social glue”, fostering social bonding, increasing mutual liking, compassion, and group cohesion^24^. It can also contribute to longer-term relationship quality, including the formation of secure attachments in caregiver-infant dyads and increased relationship satisfaction in adults^25^. This is particularly relevant in therapeutic contexts where it facilitates the therapeutic alliance^26^. Physiological synchrony, while correlated with social connection, may not always be its direct cause, often reflecting shared attention or goals, with observable byproducts mediating its detection.

Physiological synchrony refers to the temporal alignment of two or more people's physiological states^27^. In terms of cardiac activity, it can refer to the speed at which the heart beats (heart rate synchrony) or the variability of the time between heartbeats (heart rate variability synchrony, or HRV synchrony). It appears to occur spontaneously across various interperson interactions, from mother-infant dyads to psychotherapy sessions^26^. In the context of the present study, we use the term (interpersonal) synchrony specifically to denote the temporal coordination of physiological states, with a focus on heart rate variability patterns. We acknowledge that a broad range of related terms exists in the literature (e.g., adaptation, accommodation, alignment, coordination, contagion, entrainment, joint action, mimicry) and that synchrony may manifest in many processes (behavioural states such as gestures, head nods, speech, walking tempo, facial movements; physiological states including heart rate and heart rate variability, skin conductance, pupil dilation, cardiac activity and endocrine secretions; neural states involving the alignment of oscillations in neuronal polarisation, or brain-to-brain coupling; and finally affective responses like emotional contagion and regulation). However, in line with our research aims, our primary interest was to observe the mutual coordination of HRV across individuals during social interaction in the three experimental conditions. Thus, we investigate synchrony here as a pervasive feature of human social interaction, reflected in the dynamic temporal alignment of HRV across participants. This allows us to capture aspects of social connectedness that are not accessible through self-report or behavior alone, making it a powerful tool for understanding how people engage and collaborate across different contexts.

The nature and underlying mechanisms of interpersonal physio-behavioral synchrony are not entirely clear and remain a topic of debate. Representational theories propose that multimodal (behavioral, physiological, neural) synchrony arises from shared mental representations during social interaction^28–30^. In contrast, the flexible multimodal theory of synchrony emphasizes a dynamical systems perspective in which synchrony is an emergent property of interactions that requires neither central organization nor cognitive representations^22,31^. This account also allows for flexibility across modalities: behavioral alignment (verbal or nonverbal) may facilitate physiological coupling, but physiological synchrony can equally emerge independently of higher-level alignment or complementarity^22,32^. It thus highlights the dynamic, context-dependent, and non-linear nature of multimodal synchrony. In this work, however, we do not address temporal fluctuations or behavioral forms of synchrony^22^. Instead, we restrict our analysis to physiological synchrony, which we use as an index of interpersonal cohesion.

**Fig. 1:**
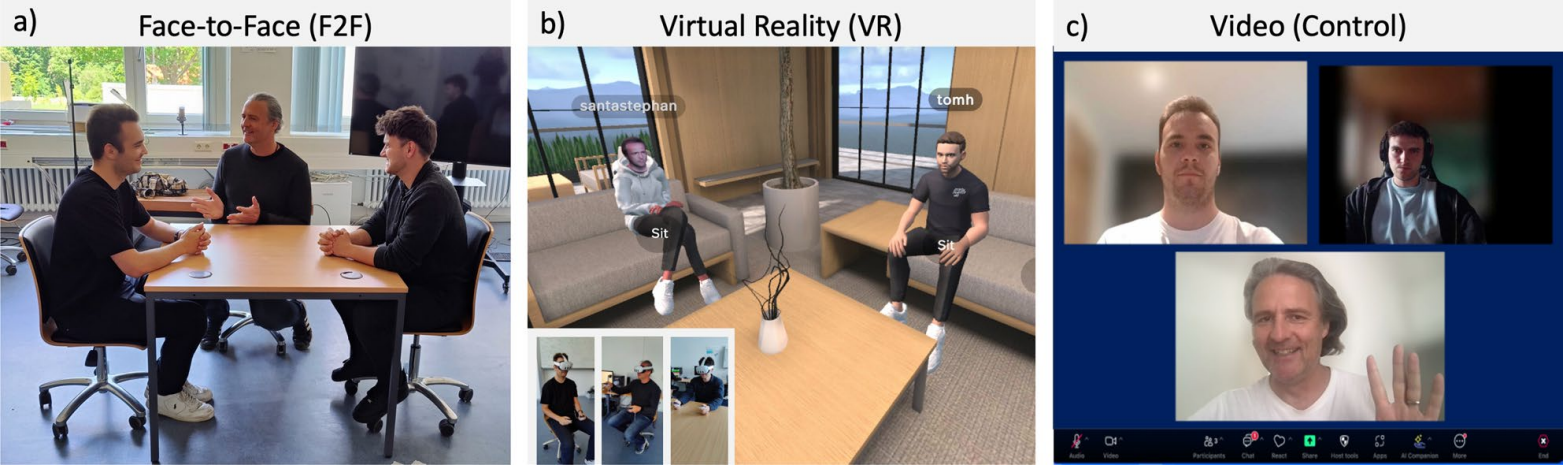
Visual depiction of the experimental setup in the different experimental conditions. In the Face-to-Face (F2F) condition (**a**), triads of participants sit together on a physical table and perform the collaborative task together. In the collaborative Virtual Reality (VR) condition (**b**), each participant is placed in a different physical room, wears a head-mounted display, and is connected to the other participants via a local network connection. Each participant is represented via a personalized avatar created from photos of the participant. Camera-based hand-tracking is used to control the avatars fingers, and the body posture of the avatar is estimated using inverse kinematics based on hand and head location. Face- and eye tracking was not possible in this setup. In the Video Condition (panel c), participants are placed in separate rooms and perform the collaborative creativity task using a video conferencing tool. In all conditions, the participants perform the same collaborative creativity task.

Previous studies have primarily examined HRV synchrony during face-to-face interactions. Thus, the question arises whether it can also occur in computer-mediated communication, which often lacks many social cues crucial for social interaction. In the current study, we aim to investigate the emergence of HRV synchrony in different media settings (VR, Video, Face-to-Face) and its implications for remote collaboration. We reasoned that different media settings would impact the interaction dynamics and synchronization processes during group interaction. Hence, the current study compared face-to-face interaction (F2F-condition) with two types of computer-mediated interaction: VR collaboration (VR-condition) and video conferencing (Video-condition) in a collaborative creativity task (see Figure 1). We measured the effect of media setting on HRV synchrony, creative group performance, and social presence. We reasoned that VR provides essential features for social interaction lacking in video conferencing, such as social presence, a shared spatial reference, and the exchange of non-verbal body-related communication cues (see Table 1 for a detailed analysis of the differences between media types). Hence, we hypothesized that groups interact more naturally and successfully in VR than in video conferencing. However, we also expect better results in the F2F condition compared to the VR condition, since the commercial VR system used in this study lacked some features that might be relevant for performing more life-like social interactions, such as face and eye tracking. Specifically, we hypothesized that:

- H1: HRV synchrony is higher in the VR condition compared to the Video condition and highest in the F2F condition
- H2: Creative group performance is higher in the VR condition compared to the video condition and highest in the F2F condition
- H3: Creative group performance correlates positively with HRV synchrony
- H4: Social Presence is higher in the VR condition compared to the Video condition and highest in the F2F condition

We tested these hypotheses in an experiment where we asked triads of participants to solve creative tasks together. We chose creative problem-solving (divergent thinking) as a collaborative task (alternative uses test). Creativity is crucial for humanity’s development and progress; therefore, it is relevant to investigate the best possible environment for creativity, not only in the individual context but also in collaboration. Previous research suggests virtual communication curbs creative idea generation^3^. However, the previously mentioned study compares group creativity scores between face-to-face and video conferencing only. Here, we include another condition to test if virtual reality collaboration produces improved group creativity scores compared to video conferencing.

## Results

### H1: HRV synchrony

**Fig. 2:**
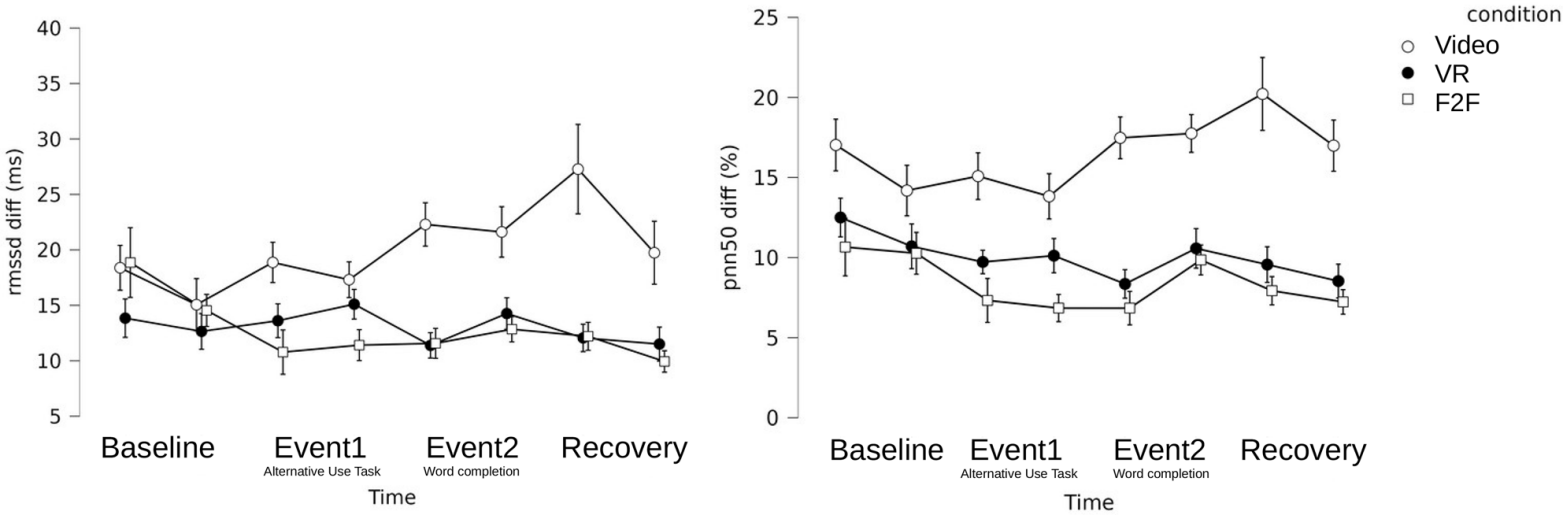
Synchrony in HRV over time, split by experimental conditions. Left panel: Changes in synchrony in RMSSD over time for every experimental condition. Right panel: Changes in synchrony in PNN50 over time for every experimental condition. Each event was split up into two equally long intervals, from which HRV values (rmssd, left panel; pnn50, right panel) were computed. Values shown are differences across participants within each triad, averaged over all triads. Thus, lower values indicate higher HRV synchrony. Error bars indicate the standard error of the mean.

To determine whether HRV synchrony varied significantly across the three conditions, repeated-measures ANOVAs were computed for HRV synchrony parameters (PNN50 and RMSSD). We employed HRV distances across the members of each group as a marker of HRV synchrony^33^. Distances were determined at 3-minute intervals to create one measure for each specified event, which were then used for the statistical analysis. This calculation was done for each group (N=23), condition, and time step (see Figure 2). Mauchly’s test for sphericity was run on all data, and correction by Greenhouse-Geisser was applied as needed. Bonferroni corrected post-hoc t-tests were computed to determine per-event comparisons.

Figure 2 shows that HRV synchrony significantly differed across experimental conditions. The lower the value, the smaller the distance between the HRV of the group members, which can be interpreted as higher synchrony of the HRV signal across members. To test for significance, repeated measures ANOVAs were run for HRV parameters, RMSSD differences, and PNN50 differences separately, with both analyses showing a significant effect for the experimental condition, with F(2,64) = 4.545, p < .05, $\eta$² = .12 for RMSSD difference, and F(2,64) = 7.882, p < .001, $\eta$² = .20 for PNN50 differences, respectively. Bonferroni corrected posthoc tests for RMSSD differences showed a significant difference between the F2F and the video condition, p < .05, and between the VR and the video condition, p < .05. There is no significant difference between the VR and the F2F condition, p> .20. For PNN50 differences, Bonferroni corrected posthoc tests showed the same pattern of results. There was a significant difference between the F2F and the Video condition, p < .001, and between the VR and the video condition, p < .01. Parallel to the results for RMSSD differences, there was no significant difference between the VR and the F2F condition, p > .20.

The result supports our first hypothesis that HRV synchrony can emerge in computer-mediated collaboration. However, the levels of HRV synchrony differed significantly across different media settings. In video conferencing, HRV synchrony was significantly reduced compared to face-to-face interaction and virtual reality collaboration.

### H2: creative group performance

Creative group performance was assessed using the Alternative Uses Test (AUT; see Gilhooly et al., 2007) and a word completion task. The AUT was evaluated using fluency, flexibility, and uniqueness. Fluency describes the number of responses, flexibility describes the number of different categories of responses, and uniqueness depicts statistically less frequent answers compared to the complete data set:

**Fig. 3:**
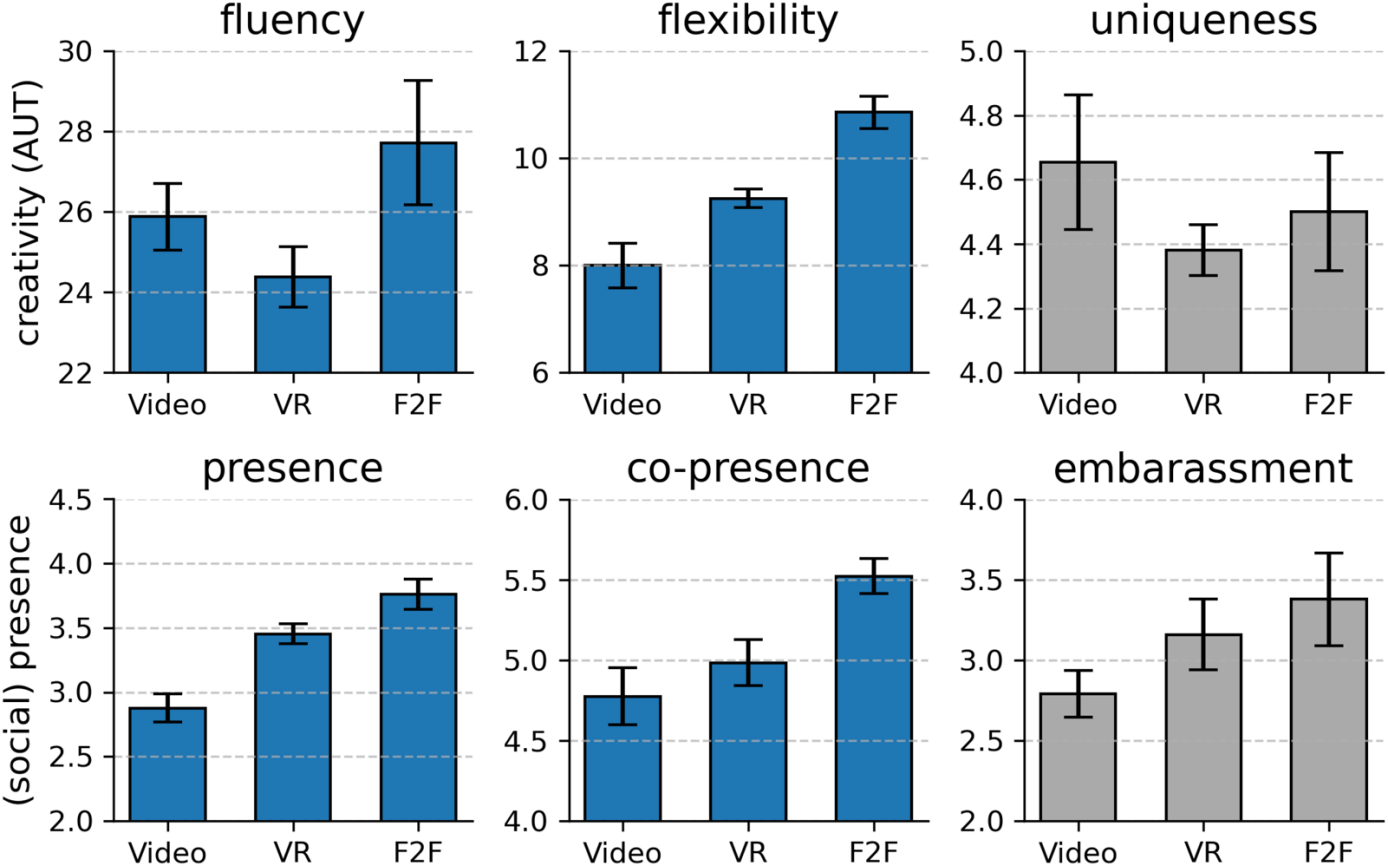
Means of AUT scores (upper panel) and (social-) presence scores (lower panel). The panels with blue bars indicate statistically significant differences between experimental conditions revealed by one-way ANOVAs. In this case, Bonferroni-corrected pairwise comparisons were applied between experimental conditions. Grey plots did not reveal significant differences, and no further analysis was used. The AUT scores (upper panel) analysis showed significant differences in the fluency and flexibility scores but no effect on the uniqueness scores. The upper left panel shows mean fluency scores. Bonferroni corrected pairwise comparisons show a significant difference between the VR condition and the F2F condition, p < .05, but no statistically significant difference between the video condition and the VR condition, p = .812, and the video condition and the F2F condition, p = .465. The upper middle panel shows mean flexibility scores. Bonferroni corrected pairwise comparisons show a significant difference between the video condition and the F2F condition, p < .001, and the VR condition and the F2F condition, p < .001. The upper right bar shows unique scores. There was no significant effect on uniqueness, p = .495. The social and spatial presence scores (lower panel) analysis showed significant differences in the fluency and flexibility scores but no effect on the uniqueness scores. The lower left panel shows the mean spatial presence scores in the different conditions. Bonferroni corrected pairwise comparisons showed a significant difference between the video condition and the VR condition, p < .001, and the video condition and the F2F condition, p<.001. There was no significant difference between the VR and F2F conditions, p = .122. The lower middle panel shows the mean co-presence scores. Bonferroni corrected pairwise comparisons show a significant difference in co-presence between the video condition and the F2F conditions, p < .001. Also, there was a significant difference between the VR condition and the F2F, p < .05. However, there was no significant difference between the video condition and the VR condition, p = .418. The lower right panel shows the mean co-presence scores and embarrassment scores. The experimental condition had no effect on embarrassment, p = .627. Note: Error bars display standard error.

#### Fluency

An analysis of covariance confirmed that gaming frequency was significantly related to fluency, F(1,63) = 7.915, p < .05, $\eta$² = .11, but group satisfaction and task satisfaction were not significantly related to fluency, F(1,63) = 1.824, p = .182, $\eta$² = .03 and F(1,63) = 2.112, p = .151, $\eta$² = .03, respectively. After controlling for the effect of gaming frequency, a significant main effect of media type on fluency remained, F(2,65) = 3.446, p < .05, $\eta$² = .10. For gaming frequency, however, the assumption of homogeneity of regression slopes was violated. Bonferroni corrected pairwise comparisons showed a significant difference between the VR condition (M = 24.38, SD = 3.68) and the F2F condition (M = 27.71, SD = 7.07), p < .05, but no statistically significant difference between the video condition (M = 25.88, SD = 4.04) and the VR condition, p = .812, and the video condition, and the F2F condition, p = .465. Figure 3 visually represents the mean group differences.

#### Flexibility

Gender was not significantly related to flexibility as a covariate, F(1,64) = 0.120, p = .731, $\eta$² = .00, but gaming frequency was, F(1,64) = 5.482, p < .05, $\eta$² = .08. For gender however, the assumption of homogeneity of regression slopes was violated. After controlling for the effect of gaming frequency on flexibility, a significant effect of experimental condition on flexibility remained, F(1,65) = 18.091, p < .001, $\eta$² =.36. Bonferroni corrected pairwise comparisons show a significant difference between the video condition (M = 8.00, SD = 2.04) and the F2F condition (M = 10.86, SD = 1.39), p < .001, and the VR condition (M = 9.26, SD = 1.39) and the F2F condition, p < .001. There was no significant difference between the video and VR condition, p = .056. Figure 3 visually represents the mean group differences.

#### Uniqueness

There is no potential covariate for the effect of the experimental condition on uniqueness. A one-way analysis of variance revealed no significant effect of experimental condition on uniqueness, F(2,66) = 0.711, p = .495, $\eta$² = .02.

#### Word completion

Analysis of covariance showed that the covariate group satisfaction was significantly related to word completion, F(1,65) = 8.529, p < .05, $\eta$² = .12. After controlling for the effect of group satisfaction, there was still no effect of experimental condition on word completion, F(2,65) = 2.394, p = .099, $\eta$² = .07.

### H3: correlation between creative group performance and HRV synchrony

To test if HRV synchrony was associated with higher group performance levels, we conducted Pearson’s correlations for the area under the curve (AUC) according to the formulas provided by Pruessner et al.^34^. The use of area under the curve measures follows the idea that by computing the average of repeated measurements, one might miss some pertinent information. The two area measures allow estimations of total activity versus time-dependent change, as outlined in^34^.

We found significant associations between the AUC of the pnn50 differences and fluency (r(67) = -.247, p = 0.044), flexibility (r(67) = -.334, p = 0.005), and uniqueness (r(67) = .252, p < 0.040). Also, there was a significant association between the AUC of the RMSSD differences and flexibility (r(67) = -.249, p = 0.042). In other words, the higher the synchrony between the group members, the lower the AUC. Therefore, the negative correlation coefficient implies that higher synchrony of HRV between the group members was linked to higher scores for the creativity measures.

We also conducted an additional correlation analysis within experimental conditions in order to outrule potentially spurious correlations. Interestingly, we found a significant correlation between HRV synchrony and fluency only in the face-to-face (F2F) condition for both parameters: RMSSD (r(23)=-0.496, p=0.016) and PNN50 (r(23)=-0.473, p=0.023). There were no other significant correlations in the other conditions nor with flexibility. It remains unclear whether the absence of significant correlations in the other conditions is due to the smaller number of data points or reflects an alternative explanation.

**Fig. 4:**
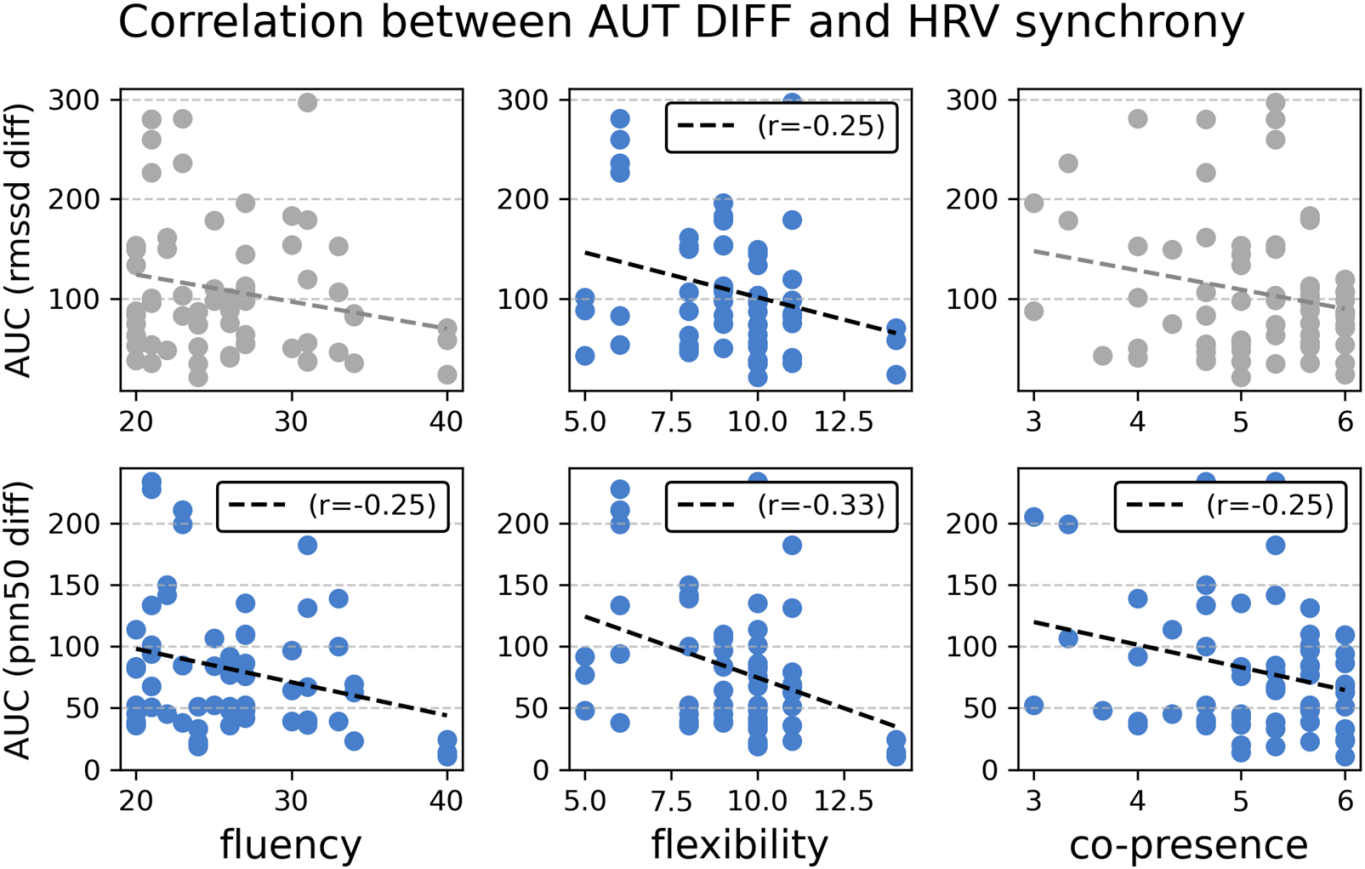
Correlations between creative group performance (fluency and flexibility) and HRV synchrony (left and middle panels) and correlations between co-presence and HRV synchrony (right panels) for both measures of HRV synchrony: the area under the curve (AUC) of rmssd differences (upper panels) and the area under the curve (AUC) of PNN50 differences (lower panels). Higher AUC scores mean larger differences in HRV and lower synchronization. Pearson’s correlations were conducted. We found significant associations between the AUC of the PNN50 differences and fluency (r(67) = -.25, p < .05), flexibility (r(67) = -.33, p < .01), and uniqueness (r(67) = .29, p < .05). Also, there was a significant association between the AUC of the RMSSD differences and flexibility (r(67) = -.25, p < .05). Furthermore, we observed significant correlations between AUC(PNN50) and co-presence (r(67) = -.25, p < .05). The negative correlation coefficients imply that higher synchrony of HRV between the group members was linked to higher scores for the creativity measures and co-presence.

### H4: social presence

We had hypothesized to observe higher levels of spatial and social presence in the F2F and VR conditions and lower levels of spatial and social presence in the video condition due to the absence of spatial and bodily cues for social interaction coordination in the video condition compared to the F2F and VR conditions.

#### Spatial presence

Analysis of covariance revealed no significant correlation between spatial presence scores and any other variable. A one-way analysis of variance revealed a significant effect of experimental condition on spatial presence, F(2,66) = 18.979, p < .001, $\eta$² = .37. Bonferroni corrected pairwise comparisons showed a significant difference between the video condition (M = 2.88, SD = 0.1) and the VR condition (M = 3.45, SD = 0.1), p < .001, and between the video condition (M = 2.88, SD = 0.1) and the F2F condition (M = 3.76, SD = 0.11), p < .001. There was no significant difference between the VR and F2F conditions, p = .122. Figure 4 shows a graphical representation of the mean group differences.

#### Co-presence

Analysis of covariance revealed a significant correlation between co-presence scores and task satisfaction, F(1,65) = 10.904, p < .001, $\eta$² = .12. After controlling for the effect of task satisfaction, there was still an effect of experimental condition on co-presence F(2,65) = 8.323, p < .001, $\eta$² = .18. Bonferroni corrected pairwise comparisons show a significant difference in co-presence between the video condition (M = 4.77, SD = 0.87) and the F2F condition (M = 5.52, SD = 0.50), p < .001. Also, there was a significant difference between the VR condition (M = 4.98, SD = 0.71) and the F2F, p < .05. There was no significant difference between the video condition and the VR condition, p = .418. Figure 3 visually represents the mean group differences.

In line with our hypothesis, presence scores differed in the experimental conditions. Presence scores were lowest in the video condition, second lowest in the VR condition, and highest in the F2F condition. However, only the spatial presence scores (ITC) produced highly significant differences between the experimental conditions.

#### Embarrassment and likeability

Analysis of covariance showed that the covariates gender (coded as 1 = male, 2 = female) and creative potential are significantly related to embarrassment, F(1,64) = 6.892, p < .05, $\eta$² =.10, and F(1,64) = 4.139, p < .05, $\eta$² =.06, respectively. After controlling for the effect of gender, with female participants scoring lower on embarrassment and creative potential, there is no longer an effect of the experimental condition on embarrassment F(2,64) = .470, p = .627, $\eta$² =.01. Further analysis of covariance confirms that the covariate group satisfaction is significantly related to likeability F(1,65) = 19.506, p < .001, $\eta$² =.23. After controlling for the effect of group satisfaction, there is no effect of experimental condition on likeability, F(2,65) = 1.010, p = .370, $\eta$² =.02. Furthermore, task satisfaction also is significantly related to likability as a covariate, F(1,65) = 17.434, p < .001, $\eta$² = .20. After controlling for the effect of task satisfaction, there is no effect of experimental condition on likability, F(2,65) = 1.949, p = .151, $\eta$² = .045.

## Discussion

This study investigated how different modes of communication affect social presence, creative group performance, and HRV synchrony during a group interaction task. A sample of 69 participants (23 triads) completed two creativity tasks in a face-to-face meeting, a video conference meeting, or an embodied virtual reality meeting. At the same time, their heart rate was continuously recorded.

The results support our hypothesis that physiological synchrony can emerge in computer-mediated collaboration. Interestingly, the levels of HRV synchrony differed significantly across different media settings. In video collaboration, HRV synchrony was significantly reduced compared to face-to-face interaction. However, HRV synchrony was not significantly different between the collaborative VR condition and the face-to-face condition, but significantly higher between the VR condition and the video condition. Given the link between HRV synchrony and group cohesion, one might suspect that only VR allows users to connect similarly to face-to-face interaction. Previous studies have shown that the absence of ”spatial faithfulness” (defined as the spatial distortions of non-verbal cues) in video conferencing can impair trust formation patterns in groups. Hence, one possible explanation for the absence of HRV synchrony might be spatial distortions of nonverbal cues in video conferencing, particularly gaze and deixis^35,36^ might negatively impact communication and synchronization patterns in video conferencing. In VR, however, ”spatial faithfulness” is preserved, allowing users to exchange non-verbal cues and social signals. VR supports deictic behaviours such as gestures, pointing and dynamic eye contact^35,36^. Hence, VR might be superior in supporting social bonding and interaction, as reflected in larger HRV synchrony in VR compared to video conferencing.

The mechanisms that allow synchrony to occur across individuals appear to be related to the ability of the autonomic nervous system to modulate cardiac activity. For example, the sympathetic and parasympathetic nervous systems can speed or slow the heart rate in response to external demands. This flexibility allows individuals to adjust their heart frequencies to match one another^27^. External stimuli, like listening to music, can also affect the autonomic nervous system, leading to augmented entrainment to temporal rhythms^37^. Since heart rate or heart rate variability are, for the most part, not directly observable, synchrony is often mediated by verbal and nonverbal cues or signals. Infants' physiological synchrony with stressed mothers can be partly facilitated by physical touch and visual stress cues. During conversations, synchronized breathing patterns also can contribute to synchronize cardiac activity. An additional motivation to align with others$'$ physiological signals might have to do with social-emotional processing. Here, the closeness of a relationship, social rapport, and shared emotions can predict heart rate synchrony^27^. For example, mother-infant dyads coordinate heart rhythms through episodes of interaction synchrony, and physiological synchrony is often investigated in the context of affect sharing and emotional co-regulation. Positive emotions can enhance temporal alignment as well. In the brain, striato-cortical loops, which are involved in representing temporal regularities, also have anatomical overlap with reward-related circuitry, supporting the coupling between regularity processing and positive affective experiences. Here, common neural systems that support both action perception and production can subtly influence individuals to synchronize their movements, potentially leading to physiological alignment^37^.

The relationship between physiological synchrony and creative group performance was also examined in this study. HRV synchrony was calculated by area under the curve (AUC) for every HRV parameter^34^ and subsequently correlated with the creative group outcome measures. A significant positive link between HRV synchrony and creative group performance was found for the AUC of PNN50 differences and fluency and flexibility and the AUC of the RMSSD and flexibility. The results align with previous studies’ outcomes, reporting a positive association between physiological synchrony and group performance^38^.

Presence refers to the psychological experience of “being there” and is a central pillar of virtual reality^39^. The current study measured spatial presence by the ITC-Sense of Presence Inventory (ITC-SOPI^40^). As expected, spatial presence was comparable in the face-to-face and embodied virtual reality conditions but significantly lower in the video conference conditions. Even though there is not much research yet on the sense of presence when attending a video conference, the findings confirm the current state of knowledge. For example, a small pilot study comparing virtual group meetings using video conferencing and immersive VR found a higher sense of presence when using an immersive VR meeting compared to a video conference meeting^13^. Another study comparing a video conference meeting with an immersive VR meeting in the financial services sector shows that participants feel significantly more present in the VR interaction than in the video interaction^12^.

Social presence, often interchangeably used with co-presence, assesses the feeling of *being there* with another person.^11^. Results show that co-presence is highest in the F2F condition and lowest in the video condition. The VR condition is placed in between. There was a significant difference between the VR and the F2F condition, but no significant difference between the video and the VR condition. Nevertheless, there is a significant difference between the video and F2F conditions, as expected. In the F2F condition, participants felt more like they were together in the room with others than in the video condition. Also, compared to the video condition, participants in the F2F condition felt more likely to be noticed by the other participants. Results of previous studies exploring co-presence in a similar context are contrary to the present results. The small pilot study by Steinicke et al.^41^ comparing virtual group meetings using video conferencing and immersive VR found higher ratings of social presence in immersive VR than in the video conference condition. Campbell et al.^42^, compared a video conference meeting with an immersive VR meeting in the financial services sector, and explored a concept comparable to co-presence, namely closeness. Participants in the immersive VR meeting felt physically closer to each other than in the video conference. Smith and Neff^43^ compared a face-to-face meeting to an embodied VR meeting with visible full-body avatars and a VR meeting without embodiment. In the embodied VR and the F2F condition, social presence was comparable. In the non-embodied VR condition, there was a significant drop in the perceived social presence. Overall, the study’s findings regarding feelings of presence support the notion that VR might be closer to reality than a video conference.

Creativity refers to “the ability to produce work that is both novel (i.e., original, unexpected) and appropriate (i.e., useful, adaptive concerning task restraints)” and is essential for everyday problem solving as well as contributing to insights or inventions in the most diverse areas^44^. This study examined creativity in the collective, i.e., group creative performance, not individual performance. Two divergent thinking tasks assessed creative group performance: the Alternative Uses Test (AUT; see^45^) and a word completion task. Previous research suggests that different properties of VR can influence creativity^46–49^. In line with previous results, we hypothesized that creative group performance is comparable in face-to-face and embodied virtual reality meetings and would drop significantly in video conference meetings. This hypothesis can only partially be confirmed. The AUT was evaluated using fluency, flexibility, and uniqueness. A significant effect of experimental conditions on fluency and flexibility was found only after including gaming frequency as a covariate. Groups whose participants play video games more often tend to have higher fluency outcomes. This aligns with previous research on the relationship between video gaming and task speed. Findings imply that video gaming might decrease reaction times without a decrease in performance accuracy.^50^ This might lead to faster responses and increased responses. However, the covariate-adjusted differences in fluency did not go in the expected direction. The F2F condition showed the highest fluency scores, followed by the video and VR conditions. Regarding the flexibility score, nearly all conditions had a significant difference. The lowest number of categories given during the AUT was found in the video condition, followed by the VR condition with the next highest score, and the highest number of different categories given was in the F2F condition. Again, gaming frequency is a significant covariate, but the effect of the experimental condition also exists without the covariate’s influence.

These findings have several implications for creativity research and the use of virtual reality (VR) in collaborative settings. First, the results suggest that VR can support social presence and physiological synchrony at levels comparable to face-to-face interactions, which are associated with enhanced creative group performance. While our study focused on divergent thinking tasks, it is plausible that similar benefits could extend to other creative contexts such as music composition, scientific ideation, or design collaboration, where coordination and synchronization are critical. Previous studies support the notion that VR can enhance creative performance in collaborative tasks such as design thinking^51^, construction of interactive restorative environments^52^, and music composition^53^. Our results shed some light on the interplay between creativity and synchronization that operate on a physiological level.

Practically, these results indicate that VR may be a viable alternative to face-to-face meetings for creative collaboration, particularly when physical co-location is not possible. VR appears to outperform video conferencing in fostering social bonding and engagement, suggesting that it should be prioritized over video for tasks that require high levels of interaction, coordination, and creative output. Video conferencing, by contrast, may suffice for less interactive or information-sharing tasks. However, it is important to further improve the technical quality of VR by including additional social cues such as eye tracking, facial tracking and realistic avatars.

## Limitations and future work

A technical limitation of our study is the absence of eye-tracking, face tracking, and full-body motion tracking, which could have enhanced the social realism of avatars in the VR condition. These technologies are critical for rendering more lifelike and realistic social interactions in virtual environments. However, due to technical and hardware constraints, most current commercial platforms and hardware do not yet support these features natively yet. Instead, they typically rely on head tracking to allow users to predict each other’s gaze direction based on head orientation, and they use inverse kinematics algorithms to estimate body posture and movements based on limited input (e.g., headset and controller positions). Despite these limitations, such systems still enable key aspects of social interaction, including turn-taking and basic gesturing, and can evoke a sense of social presence. Our findings suggest that even with these constraints, current social VR platforms are sufficient to elicit physiological synchrony patterns comparable to those observed in face-to-face communication, and notably greater than those seen in video conferencing contexts.

However, the social presence and creativity scores in our study showed significantly better results in the F2F condition compared to the VR condition, indicating a lack of social realism in the VR condition. Recent studies implemented face- and eye tracking in immersive multi-user VR^14,54,55^. These studies showed gaze and speaking patterns that closely follow patterns known from face-to-face interactions^14,54^ and different patterns observed during video conferencing^55^. We can only speculate how the inclusion of eye and face tracking would affect our result, but it is likely that it would decrease the observed differences between F2F and VR conditions and would increase the differences between VR and video condition. Future studies need to verify this assumption.

Using full-body tracking in the different experimental conditions would have also allowed us to analyze the relation between physiological and behavioral (movement) synchrony. However, this was beyond the scope of our current study. It should be addressed in future experiments.

One possible explanation for the relative absence of physiological synchrony in the video condition is that participants might have moved less in this condition compared to the VR and F2F conditions. Since we did not collect motion capture data for the F2F or video conditions, we cannot empirically test this hypothesis in the current study. Nonetheless, we suggest that future research systematically examine the interplay between behavioral and physiological synchrony, including how behavioral coordination might reinforce or drive physiological synchrony.

In the current study, the creative task was purely verbal and did not involve any form of object manipulation. In many real-world collaborative settings, teams interact verbally and physically with shared objects (e.g., designing a car together). This physical component may introduce different coordination dynamics and synchronization patterns not captured in the present study. Future work should investigate if multi-user object manipulation in VR enhances HRV synchrony in various tasks, such as stacking blocks^56^ or carrying an object together^57^.

A potential limitation of our study is the relatively small sample size, with N = 23 groups of three participants each.

A post hoc power analysis was conducted to assess the sensitivity of our repeated-measures ANOVA, which included a between-subjects factor (3 group conditions) and a within-subjects factor (8 time points), with the 69 subjects as the unit of analysis. Assuming a medium effect size (f = 0.4), an alpha level of 0.05, and a nonsphericity correction $\epsilon$ = 1, the estimated statistical power for detecting a main effect of group was approximately 0.83 (https://webpower.psychstat.org/models/means05/). This suggests that the study was powered to detect medium-sized effects, but perhaps underpowered to detect smaller than medium-sized effects. Consequently, a future study with a larger sample size could reveal differences in HRV synchrony between the VR and F2F conditions. Nevertheless, the design was robust enough to detect significantly greater HRV synchrony in the VR and F2F conditions compared to the video condition.

Additionally, while our study focused on physiological synchrony, incorporating behavioral synchrony measures may provide a more comprehensive understanding of the dynamics involved in the experimental task.

## Conclusion

This study compares physiological synchronization processes during collaborative problem-solving in real life (face-to-face condition) with two computer-mediated conditions using video conferencing (video condition) and virtual reality (VR condition). We measured the effect of media type on heart rate variability (HRV) synchrony, divergent thinking, and social and spatial presence. We found evidence for stronger HRV synchrony in face-to-face and VR conditions compared to the video condition. Weak physiological synchrony in the video condition is associated with lower group performance and social presence. However, we also observed lower social presence and creativity scores in the VR condition compared to the F2F condition. Improving the technical setup of the VR platform used in this experiment by including face and eye tracking might further increase social realism and render the observed differences between VR and F2F condition smaller. Future studies need to investigate this. The lack of physiological synchrony in video conferencing might be interesting concerning the underlying physiological mechanism of the novel phenomenon of “Zoom Fatigue”. This idea needs to be examined in future studies, and it also needs to be determined whether the effect goes beyond the context of creative group tasks. Finally, we used HRV synchrony as a marker of social cohesion. HRV synchrony is easy to measure and calculate, and provides an interesting opportunity to capture social interaction parameters in computer-mediated communication settings.

## Methods

### Participants

Sixty-nine healthy students enrolled at the University of Konstanz were recruited via an online recruitment platform. Participants were randomly assigned to one of the three experimental conditions and participated in three groups. The mean age of the total sample was 22,9 years (SD = 3,4 years), ranging from 18 to 37 years. Participant exclusion criteria were previous cardiac and neurological conditions, which might interfere with heart rate variability parameters. Before the experiment, participants were informed of the study procedure and gave written informed consent. Participants received financial compensation (15€) or experiment participation credits after the experiment. The study was approved by the ethics committee at the University of Konstanz (IRB 03/2019), and it was performed in accordance with the Declaration of Helsinki. Written informed consent was obtained from all participants prior to the experiment. Written informed consent for publication of identifying images was obtained from all persons depicted in this article.

### Experimental design

A between-group design with three experimental conditions was employed to compare the effect of communication medium on HRV synchrony, social presence, and creative task solving in triads of participants. In the F2F condition, triads performed the task in an unmediated face-to-face situation, where participants were seated together at a table. In the video condition, all three participants were placed in separate rooms and performed the task using the Zoom video conferencing tool (https://zoom.us/). Participants were placed in separate rooms in the VR condition and performed the task using the collaborative VR application Spatial (https://www.spatial.io/). In this condition, each participant wore a head-mounted display (Oculus Quest 1) to be immersed in a shared virtual office. Each participant could hear the other participants through the headphones of the head-mounted display. Furthermore, each participant was embodied as a virtual avatar animated in real-time by the user’s body movements. This allowed participants to look at each other and to perform gestures and pointing movements using their virtual hands. Figure 1 illustrates the setup and characteristics of the three experimental conditions.

**Table 1:** Comparison video conferencing, virtual reality and face-to-face meetings.

	Video	VR	Face-to-face
Verbal cues	Yes	Yes	Yes
Non-verbal cues	Face	Face+body	Face+body
Joint attention	No	Yes	Yes
Spatial presence	Low	High	High
Co-presence	Low	High	High
Computer-mediated	Yes	Yes	No

### Task

In all three conditions, triads were asked to perform three consecutive tasks. In the first task, participants were asked to exchange their opinions about the local city (Constance, Germany) with each other to get familiar with the group. This task was used as a warm-up for the group and was not included in the analysis. The second and third tasks were designed to assess the group’s divergent thinking ability as a measure of creativity. The group was challenged with an Alternative Uses Test (AUT) and a word completion task. Both tasks can be classified in the McGrath task circumplex into the first quadrant “to Generate”, characterizing tasks where the dominant coordination process requires the group to generate new ideas and information and therefore aims at measuring creative group performance^58^. Both tests required participants to work together to find creative solutions.

### Procedure

Laboratory sessions took place at the University of Konstanz between 9:30 am and 5:00 pm and lasted approximately 90 minutes. The same experimenter conducted all sessions. After providing written consent, participants were asked to put on a heart rate sensor (Polar H7, Finland) that continually recorded their heart rates throughout the session. Participants were then given a paper-and-pencil questionnaire to collect demographic data and assess their creative potential using the Creative Achievement Questionnaire (CAQ^59^. After completing the questionnaire, participants were asked to remain seated quietly for at least five minutes to allow for a baseline measurement of HRV. During baseline measurements, participants were seated in different rooms and could not see or interact with one another. Afterward, participants were greeted in person, in the Zoom call, or in the VR environment, and a short interaction task was given to the triad so that participants could get to know each other a little. Afterward, triads had 5 minutes to collaboratively conduct the Alternative Uses Test (AUT; based on^60^). Subsequently, a word completion task was presented to the triad, and here, as well, the participants had five minutes to work on it together. In the face-to-face condition, the tasks were handed out in a paper-and-pencil format; in the mediated conditions, the experimenter provided the tasks via screen-sharing. Afterward, participants were given a paper-and-pencil closing questionnaire that assessed their feelings of spatial presence, co-presence, and satisfaction with the group and the task completion. After completing the closing questionnaire, participants were again asked to remain seated quietly for at least five minutes to allow for a recovery baseline measurement of HRV. At the very end, a debrief document was handed out in which the purpose of the study was explained in more detail. There was also space for comments, remarks, or questions. Contact details were provided so participants could contact us with any follow-up questions.

**Fig. 5:**
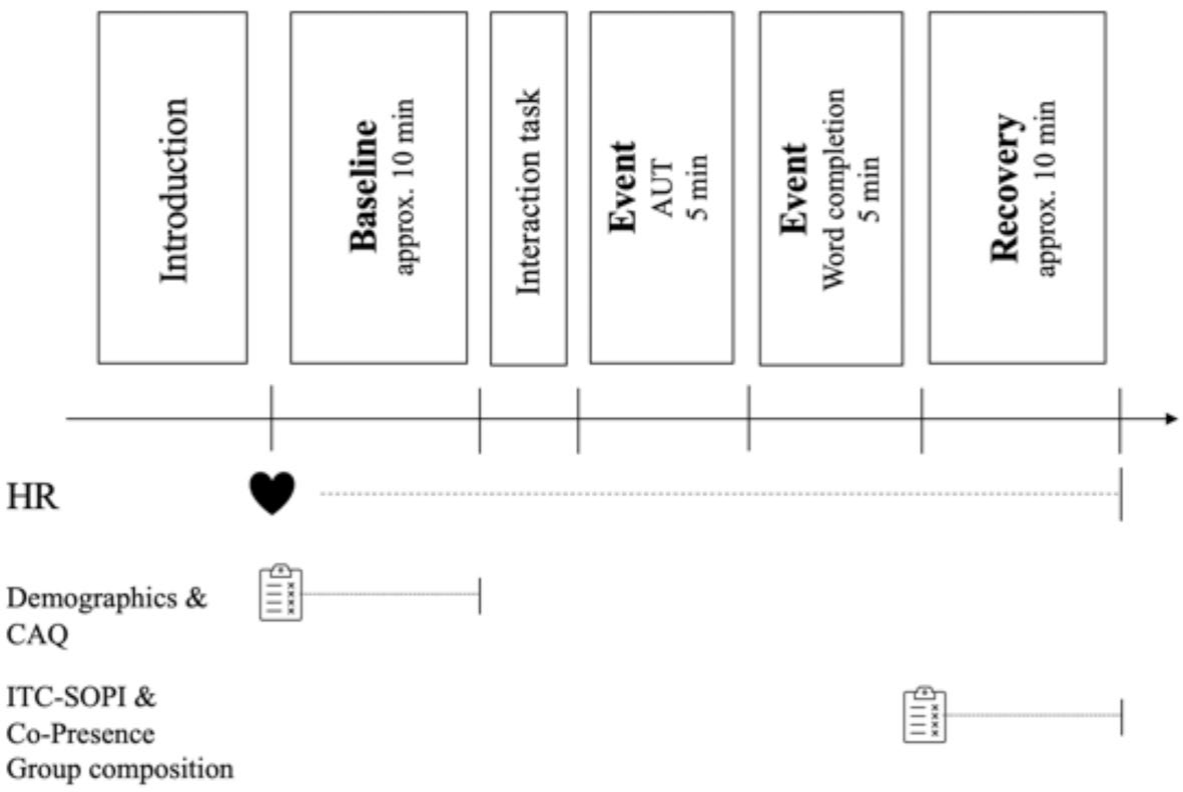
General experimental procedure: note. *CAQ* creative achievement questionnaire; *ITC-SOPI* ITC-Sense of Presence Inventory. Vertical dividers on the timeline represent placed markers for the analysis of HRV synchrony.

### Data analysis

Data analysis was conducted using JASP version 0.14.1 (JASP Team, 2024). First, to discover potential co-variates, bivariate Pearson’s correlations were calculated between the dependent variables (spatial presence [ITC score] and co-presence as specified by co-presence, embarrassment, and likeability) and the variables age (in years), gender, creative potential (CAQ score), gaming frequency, group satisfaction, and task satisfaction. If any of the variables correlated significantly with a dependent variable, ANCOVAs were calculated to control for the covariate’s influence when investigating whether there is a difference between conditions. Otherwise, one-way ANOVAs with the experimental conditions as an independent variable were performed for the ITC score and every co-presence measure. For both significant ANOVAs and significant ANCOVAs, Bonferroni-corrected pairwise comparisons were applied to locate group differences.

### Measurements

#### Personal data

Demographic data, including age, gender, and study major, were collected. Participants were also asked about their experience with video conferencing, computer and video games, and virtual reality systems.

#### Heart rate variability (RMSSD and PNN50)

Individual heart rate variability (HRV), the time intervals between two subsequent heartbeats in milliseconds in a participant, the RR intervals, were measured using Bluetooth low-energy Polar H7 heart rate sensors (sampling rate: 1,000 Hz) mounted on a two-electrode chest strap (Polar, Finland) in combination with the application Heart Rate Variability Logger for iOS (Altini, 2013) running on an iPad (Apple Computer, Cupertino, CA, USA).

After the recording, raw data was transferred to a personal computer, where further processing was done using the software program *R* (R Core Team, 2024, version 4.4.1, https://www.r-project.org/). Within R, we first identified and removed outliers using in-house scripts that combine automated algorithms and visual inspection to identify and remove movement artefacts and ectopic beats. To maintain the overall integrity (i.e., recording length and time dynamics) of the ECG for synchronization analysis, the script then interpolated missing values using best-guess estimates from surrounding intervals. Less than 5% of missing values were replaced using best guess estimates. HRV was then calculated by defining various length intervals and computing the root mean square of successive differences (RMSSD) using the RHRV package within R^61^. PNN50 was calculated as the percentage of successive R-R interval differences that are greater than 50 ms^61^. The preprocessing and calculation procedure of RMSSD and PNN50 follows Geurts at al. 2023.

RMSSD and PNN50 are standardized markers of HRV derived from time-based analysis. Both are commonly reported and accepted. While RMSSD reflects overall beat-to-beat variability and is considered a robust index of parasympathetic activity, PNN50 specifically quantifies the proportion of larger successive differences (>50 ms), making it more sensitive to pronounced fluctuations in heart rate.

#### Heart rate variability synchrony

Several types of HRV synchrony measures exist, including temporal coupling metrics (e.g., windowed cross-correlation), spectral or phase-based metrics (e.g., wavelet coherence, phase-locking), and nonlinear measures (e.g., cross-recurrence, mutual information). Because our interest lies in shared autonomic activation states during discrete events, we employed distance-based HRV synchrony, calculated as the mean pairwise HRV distance across group members in three-minute windows. This approach captures state similarity rather than moment-to-moment coupling and provides an interpretable, event-level synchrony index well suited to our research question.

To calculate HRV synchrony for each group, HRV parameters were first calculated separately for each participant (see 1). During the experiment, markers were set in the HRV Logger application (Altini, 2013) to divide the experiment into meaningful subsections. Set marker timing can be observed in Fig. 5 (denoted as vertical lines), resulting in four events: baseline, AUT, word completion, and recovery. For analysis, an 8-minute window of each participant’s baseline was used, a 5-min duration of the AUT, a 5-minute duration of the word completion task, and an 8-minute window of each participant’s recovery baseline. All events were split into two parts, allowing for a higher temporal resolution. This resulted in eight RMSSD computations for each subject: two baseline events (after 4 and 8 min), two AUT events (after 2,5 and 5 min), two-word completion events (after 2,5 and 5 minutes) and two recovery events (after 4 and 8 min). To compute synchrony, the absolute distance of RMSSD and pnn50 scores across all group members were calculated at each point in time to allow for a continuous marker of synchrony, which could also be employed in a repeated measures analysis to capture dynamics over time. This way, the absolute distance and the conversion toward each other, or the divergence of group members over time, are captured. This approach is similar to the computation of spike train distance for the computation of neuronal synchrony^62^. It assumes neither the individual RR interval nor the overall activity level is most relevant. However, looking at the RR stream as a spike train, the information maintained is the timing of the individual spikes (RR intervals), which are the basis for RMSSD calculations. Thus, similar or converging RMSSD scores over time indicate a form of synchrony. ECG recordings were unusable in 2 participants (more than 20% of erroneous data). They thus had to be excluded from the analysis, one from the video condition and one from the VR condition. This resulted in HRV analysis based on n = 67 subjects.

#### Creative group performance (divergent thinking)

To assess the group’s divergent thinking ability as a measure of creativity, the groups were presented with two tasks: An Alternative Uses Test (AUT) and a word completion task. Both tasks can be classified in the McGrath task circumplex into the first quadrant “to Generate”, characterizing tasks where the dominant coordination process required the group to generate new ideas and information and therefore aim at measuring creative group performance^58^.

The Alternative Uses Test (based on Guildford, 1967^63^) is a standard divergent thinking test with substantiated reliability and validity. The test is about finding as many alternative use cases as possible for a known object beyond its conventional use. Participants were given five minutes to think of as many alternative uses for a brick as possible, whilst the experimenter recorded the results. The responses are rated on three components: fluency, uniqueness, and flexibility. Within the framework of creativity research, fluency denotes the capacity to generate a large number of ideas, flexibility refers to the ability to shift across diverse categories or perspectives, and uniqueness captures the degree of novelty or statistical infrequency of the responses. While fluency and uniqueness are relevant to divergent thinking, flexibility, reflecting the ability to generate ideas across diverse categories or perspectives, is considered the most critical factor for creativity, as it underlies the cognitive adaptability necessary for producing truly innovative solutions.

To analyze uniqueness, the response frequency in the complete sample was counted (naming frequency). Subsequently, the naming frequencies of all group responses were averaged, the value constituting the originality score of a group. The higher the originality score, the less original the group’s ideas. The *number of different categories* of alternative uses depicts the flexibility component. To classify responses, the experimenter developed a classification system and cross-checked by a second, independent person. To the best of current knowledge, no norm values and categories are available for the brick as an object of the AUT. Therefore, the uniqueness and flexibility of the answers were evaluated within the present sample and not based on norm values. Nevertheless, the evaluation was oriented towards the established scheme of previous studies that used a paper clip as an object for the AUT^64,65^. Echoing previous studies, the fourth possible component to analyze the AUT, namely elaboration, was not assessed in the present study.

In the word completion task, 50 words were randomly taken from a list of the 500 most common words in the German language. Letters were then erased from each word and marked as *gaps*. Participants were given five minutes to fill the gaps with the correct letters and thus complete the words while the experimenter recorded the results. For each correct word, the group was given one point.

#### Spatial and social presence

Spatial Presence is defined as the ‘feeling of being located within a virtual space’^39^. It was assessed by the spatial presence subscale ($\alpha$ = .93) of the ITC-Sense of the Presence Inventory (ITC-SOPI), a self-report instrument^40^. The ITC-SOPI is a cross-media questionnaire aimed at measuring presence across various settings such as 2D and 3D environments, movies, or video games and, therefore, is suitable for the present study.

Co-presence represents the degree to which participants perceive and treat other group members as real people^11^. We used the questionnaire provided by Bailenson et al.^11^ to measure co-presence. The questionnaire utilizes statements like *“I felt like there was someone else in the room with me”*. Perceived co-presence was measured with three items and compounded to a co-presence measure by averaging responses ($\alpha$ = .71). Higher scores indicated higher levels of self-reported co-presence.

Embarrassment was assessed as a self-reported social response to the other group members. It has been shown in earlier research that the willingness to perform embarrassing acts in front of virtual social agents is a measure of the agents’ social influence^11^. Thus, the more agents are perceived as artificial, the more willing participants are to perform embarrassing acts in front of them. Three statements regarding embarrassment were made, and the responses were averaged into a single measure ($\alpha$ = .72), with a higher score indicating a higher willingness to perform embarrassing acts in front of the other participants. One exemplary statement is *“I would be willing to change clothes in front of the other group members”*.

Likeability was assessed with an average score of four likeability statements ($\alpha$ = .71), with a higher score indicating a higher liking of the other group members by the participant. One statement, for example, was *“I would like to meet the other group members again”.*

#### Creative achievement questionnaire

The Creative Achievement Questionnaire (CAQ^59^) was used to assess participants’ past creative achievements across ten domains of creativity to control for possible baseline differences in creative potential. Higher scores indicate more creative experiences and, hence, higher creative potential. The German translation by Form et al. (2017) was used for the present study.

## Data Availability

The data are available and can be shared upon reasonable request. Please contact the corresponding author via email for further information.
